# Symmetry Function: The Differences between Active and Non-Active Above-the-Knee Amputees

**DOI:** 10.3390/s22165933

**Published:** 2022-08-09

**Authors:** Mateusz Kowal, Sławomir Winiarski, Ewa Gieysztor, Anna Kołcz, Ilias Dumas, Małgorzata Paprocka-Borowicz

**Affiliations:** 1 Department of Physiotherapy, Wroclaw Medical University, 50-367 Wroclaw, Poland; 2 Department of Biomechanics, University School of Physical Education in Wroclaw, 51-612 Wroclaw, Poland; 3 Ergonomics and Biomedical Monitoring Laboratory, Wroclaw Medical University, 50-367 Wroclaw, Poland

**Keywords:** symmetry, time series, gait, transfemoral amputation, physical activity

## Abstract

The number of patients with unilateral above-knee amputation (AKA) due to non-vascular causes has remained stable over the years, at 0.92 per 1000 people per year. Post-AKA individuals are at risk of experiencing a higher incidence of chronic pain. Post rehabilitation, it is estimated that between 16–62% of patients with musculoskeletal disabilities fail to meet the minimum criteria for physical activity in comparison to a healthy population. The current study included 14 participants (11 men and 3 women) with a mean age of 46.1 ± 14.2 years, body height of 1.76 ± 0.09 m, and weight of 79.6 ± 18.3 kg, who were all post-unilateral above-the-knee amputees. Patients in the study were divided into two groups: active (AC) and non-active (NAC). This study was conducted in a certified Laboratory of Biomechanical Analysis using the BTS Smart-E system (BTS Bioengineering). In order to investigate the symmetry function (SF) of gait, the only measurements included were the time series assessment of gait variables defining pelvic and lower limb joint motion and ground reaction forces (GRF). Both groups had an asymmetrical gait pattern with a different magnitude and relative position in the gait cycle, which was revealed by SF. The differences in terms of median, minimum, and maximum were statistically significant (*p* < 0.05), with SF ranging from –25 to 24% for the AC group and from 43 to 77% (59% on average) for the NAC group. The AC’s pattern was more symmetrical compared to the NAC’s pattern, especially in the case of pelvic and hip joint motion.

## 1. Introduction

The number of individuals with unilateral above-the-knee amputation (AKA) due to non-vascular causes has remained stable over the years, at 0.92 per 1000 people per year [1,2]. The cornerstone of successful rehabilitation after AKA is prosthesis implantation as early as possible to restore gait function [3]. Despite significant advances in prosthetics, AKA amputees are more likely to experience pain than non-amputees are. The above-mentioned pain is usually chronic. Chronic pain is recognized by the WHO as a disease and is one of the most prevalent diseases worldwide, resulting in substantial disability [4]. Chronic pain is defined as recurrent or persistent pain that lasts beyond the normal time of healing (usually more than 3 months) [5]. Causes of chronic pain in AKA amputees include gait asymmetry, phantom limb pain (defined as a painful sensation below the amputation) [6], neurogenic pain, limb joint pain (due to accelerated degenerative changes in osteoarthritis (OA), and lower back pain [7,8]. A typical response to chronic musculoskeletal pain is a motor adaptation that protects the injured body part. Motor adaptation enables the body to function while causing more asymmetry between the left and right sides of the body. Asymmetry between limbs exacerbates gait asymmetry [9]. According to studies, chronic pain affects the gait cycle: the majority of changes described include decreased gait speed [10], altered pelvic and knee joint ranges of motion, and limb loading asymmetry [11,12].

One way to reduce the development of chronic pain or counteract its negative effects is by physical activity (PA) [13,14]. PA is defined as “any bodily movement produced by skeletal muscles that require energy expenditure” [15]. According to WHO recommendations, PA for those with musculoskeletal disabilities should be 150 min of moderate-intensity and vigorous-intensity activities throughout the week (i.e., >3 times the intensity of rest) [16]. It is estimated that 16–62% of people with musculoskeletal disabilities do not meet these PA criteria compared to healthy individuals [17], even though AKA amputees appear to benefit both physically and mentally from playing sports and/or regular PA during rehabilitation [18,19]. PA also affects the degree of pain experienced [10,20,21,22,23,24]. Reports indicate a correlation between gait parameters and the muscle strength moment of the muscles acting on the hip joints of AKA amputees [25]. Our previous studies demonstrate different forms of gait adaptation in people after AKA depending on the knee prosthetic modules used [26]. For this reason, the hypothesis was adopted that PA influences the reduction of gait asymmetry in AKA amputees. Moreover, the following research questions were formulated: What are the largest values of asymmetry in terms of ranges of motion and limb loading? In which phase of the gait cycle do they occur in AKA amputees? Do physically active AKA amputees exhibit greater symmetry of range of motion within the pelvis and lower extremity joints than non-active individuals? Will PA affect the ratio of the motion range and ground reaction force between limbs in AKA amputees?

## 2. Materials and Methods

### 2.1. Participants

This was an observational study with convenience samples. The study included 14 participants (11 men and 3 women) with a mean age of 46.1 ± 14.2 years, body height of 1.76 ± 0.09 m, and weight of 79.6 ± 18.3 kg; all participants were post-unilateral above-the-knee amputees. The patients were divided into two groups: active (AC participants in the active group took part in sports activities at least 3 times a week for 2 h) and non-active (NAC). The detailed characteristics of the two groups regarding orthopedic supplies and the type of activity they engaged in are shown in Table 1. All participants used their prostheses on a daily basis (for a minimum of six months before the study) and did not use any other walking aids. The inclusion criteria included the Medicare Functional Classification Level: K-level 3 or 4. Subjects in the study group used the prosthesis on a daily basis for at least 9 h. Prior to the start of the measurements, all participants declared that on the day of the measurements, they had no pain in the thigh stump or the sound limb. The exclusion criteria included (1) medical history of musculoskeletal injuries causing pain, weakness, decreased range of motion, and coordination loss, (2) dysfunction of the neuromuscular, cardiovascular or respiratory systems, and (3) pain in the area of the stump or lower limb.

### 2.2. Experimental Protocol and Setup

**Figure 1:**
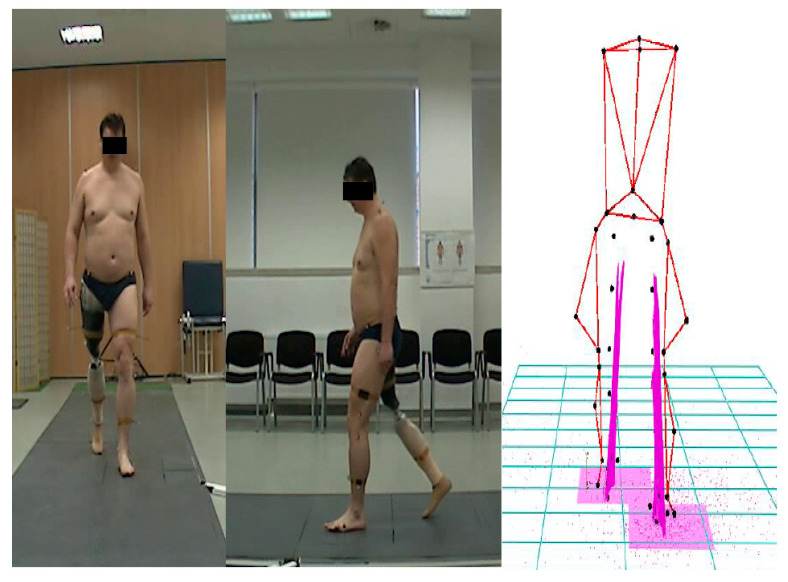
Measurement of gait kinematics and kinetics. Front and side view and model projection created from marker tracking by BTS System.

This study was conducted in a certified Laboratory of Biomechanical Analysis (PN-EN ISO 9001:2001) using the BTS Smart-E system (BTS Bioengineering, Milan, Italy). The cameras used in this system were: six infrared digital cameras (1.1 μm infrared light, 120 fps in 768 × 576 px resolution); two Network Cam AXIS 210A cameras (AXIS Communications, Tokyo, Japan) operating within visible range at 20 Hz; two piezoelectric force plates type 9286A (at 1 kHz; Kistler Instrumente AG, Winterthur, Switzerland) designed specifically for use in gait analyses with a measurement threshold of more than 250 mN and a range of 0–5 kN for the vertical component. Twenty-two photoreflective film markers were placed on the subject’s body following the procedure for the modified Hayes–Davis model. (Figure 1). 

In the adopted gait model, the gait cycle consisted of eight gait phases: initial contact, loading response, mid-stance, terminal stance, pre-swing, initial swing, mid-swing, and terminal swing [27]. The measurements involved in testing symmetry function (SF) were limited to evaluating the time series of gait that determine pelvic motion and the motion of lower extremity joints. Following the ISB recommendations on definitions of the joint coordinate system for the reporting of human joint motion [28,29], the following angles were chosen, sampled at 120 Hz and normalized to cycle time (% CT):

- pelvic obliquity (PO): upward (positive) or downward (negative) pelvic motion in the frontal plane;
- pelvic tilt (PT): anterior (positive) or posterior (negative) pelvic motion in the sagittal plane;
- pelvic rotation (PR): internal (positive) or external (negative) pelvic motion in the transverse plane;
- hip abduction/adduction: positive (abduction) or negative (adduction) movement of the femur in the frontal plane;
- hip flexion/extension: positive (flexion) or negative (extension) movement of the femur in the sagittal plane;
- hip rotation: internal (positive) or external (negative) movement of the femur in the transverse plane;
- knee flexion/extension: positive (flexion) or negative (extension) movement of the femur in the sagittal plane;
- ankle dorsiflexion/plantarflexion: positive (dorsi) or negative (plantar) movement of the foot with respect to the tibia in the sagittal plane.

Three ground reaction force (GRF) components were also analyzed: vertical, anterior-posterior (AP), and mediolateral as a function of normalized time (% of stance time, % ST). The GRF components were normalized to body weight and expressed as a percentage (% BW).

### 2.3. Data Analysis

All individual gait cycles were averaged first within and then between participants. To investigate symmetry, dynamic symmetry function (SF) was used. In its general form, SF is a function of time (t) and expresses the percentage difference between the involved XIN and uninvolved XUN sides relative to an average range of change.

$$SF(t)=2\cdot \frac{x_{in}\left(t\right)-x_{un}\left(t\right)}{Range\left(x_{in}(t)\right)+Range\left(x_{un}(t)\right)}.$$

SF function can be interpreted in a similar way to the symmetry index (SI), with SF values < 5% indicating good symmetry, values between 5–10% indicating moderate symmetry, and values > 10% indicating asymmetry. Positive values indicate a predominance of the involved limb over the uninvolved one [30]. 

For each participant and each of the measured cycles, time normalization of the right and left angles for the sagittal plane or GRF components was performed numerically, using decomposition of a time series through the Lagrange interpolation polynomial. This way, right and left cycles of the same length (100% CT for angles and 100% ST for GRF components) were obtained. Moreover, the graphs of the range of motion in joints and SF function were parameterized to compare values between groups. Key values were extracted for each graph, separately for the left and right sides and the resulting SF function. These values included maximum (Max) and minimum (Min) values, range of change (Range), median value (Median), and median absolute deviation (MAD). The Median determines the central tendency of the data sets. The MAD is the equivalent of a standard deviation (SD) and is a measure of spread that represents expected absolute error loss and is more robust to outliers. The MAD is a measure of variability and dispersion of data points around the Median value. The extraction of the points of interest was conducted before averaging.

### 2.4. Statistical Analysis

Due to the small size of the compared groups, all the analyses were performed with the use of nonparametric tests. The basic descriptive statistics (medians and quartiles (Q1 and Q3)) were evaluated for the extracted peak values and their locations in the gait cycle (Min, Max) and ROM. The nonparametric Wilcoxon signed-rank test was used for testing the differences between dependent parameters for the involved and uninvolved sides and between events for different profiles of angles and SF function (α = 0.05). The paired Wilcoxon was used for comparing all variables between active and non-active participants and between the involved and uninvolved side knees in each group. The relationships between static and dynamic postural stability and knee muscle strength were assessed using the Spearman coefficient of correlation (r) for each group. All levels of statistical significance were set at α < 0.05. All statistical analyses were performed using Statistica 13.1 (TIBCO Software Inc., Palo Alto, CA, USA). Power analysis was conducted to determine the sample size, with a power of 0.8 and an α level of 0.05. A pilot study with 5 knees in each group indicated that 42 knees would be required to detect any significant differences. The power for detecting between-group differences in postural stability was 0.804 in this study.

## 3. Results

The average intraclass correlation coefficient (ICC, intra-individual variability) for a test–retest of three repetitions of walking, based on extracted parameters and measured during three different periods, was ICC = 0.91, with a 95% confidence interval (5% margin of error). The average standard error of measurement (SEM) was 0.41 with an acceptable level of reliability r = 0.9, which shows good repeatability and reliability. The mean speed registered for the active group (AC) was 0.99 ± 0.12 (m/s) and 0.91 ± 0.08 (m/s) for the non-active group (NAC), with no statistically significant differences between the groups (*p* < 0.05). Both groups had an asymmetrical gait pattern with a different magnitude and relative position in the gait cycle revealed by SF. 

**Figure 2:**
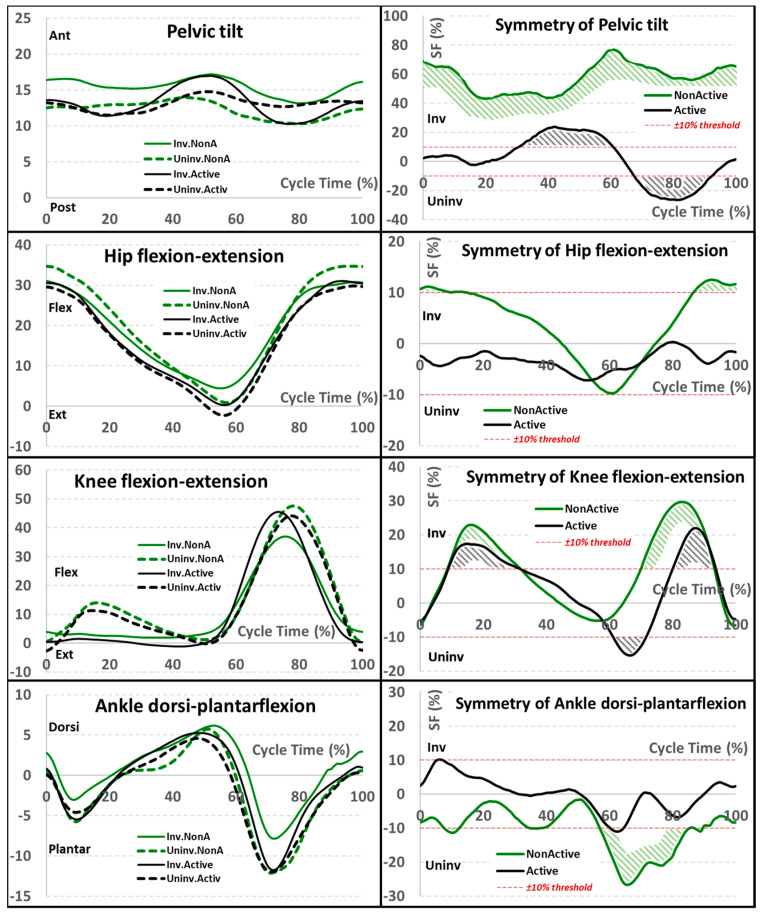
Sagittal plane kinematics for the pelvis, hip, knee and ankle joints (in degrees) for both involved/uninvolved limb and active/non-active groups (**on the left**); corresponding symmetry function (SF) for the active/non-active groups (**on the right**). The area exceeding the ±10% threshold was highlighted to discriminate between symmetrical and asymmetrical patterns. All angles are in degrees and the SF in percentage.

For the sagittal plane (Figure 2, Table 2), the asymmetry areas (area for SF exceeding the established ±10% level) were found primarily in the pelvic tilt during terminal stance (40–60% CT), the mid-swing (70–90% CT) for the AC group, and during the whole pelvic motion for the NAC group. The differences in terms of median, minimum, and maximum values were statistically significant (*p* < 0.05), with SF ranging from −25 to 24% for the AC group and from 43 to 77% (59% on average) for the NAC group (Table 2). The motion of the hip was rather symmetrical except for initial contact (5% CT) and terminal swing (90–100% CT) for the NAC group with 12.5% SF. The ankle dorsiflexion/plantarflexion was relatively symmetrical for the AC patients within the adopted ±10% level but asymmetrical during the swing phase for the NAC patients. The highest SF value was −27% due to the predominance of the uninvolved limb over the involved one (0.5 vs. −11.9 degrees of plantarflexion at swing).

**Figure 3:**
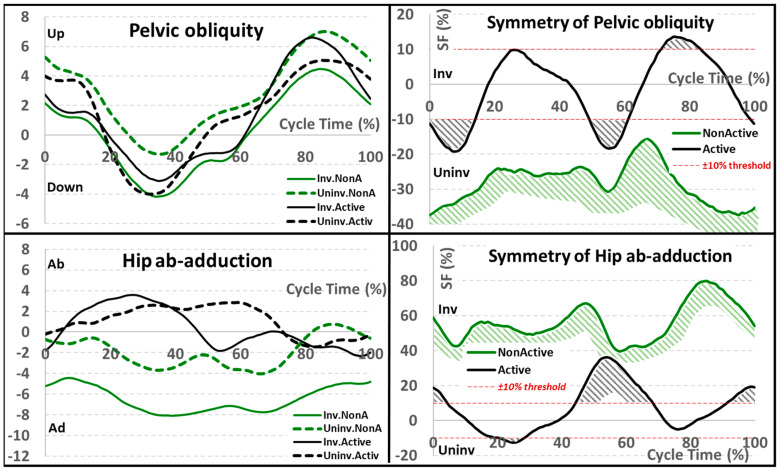
Frontal plane kinematics for pelvis and hip joints (in degrees) for involved/uninvolved limb and active/non-active groups (**on the left**); corresponding symmetry function (SF) for the active/non-active groups (**on the right**). The area exceeding the ±10% threshold was highlighted to discriminate between symmetrical and asymmetrical patterns. All angles are in degrees and the SF in percentage.

For the frontal plane (Figure 3, Table 2), non-active subjects showed an asymmetrical pelvis pattern in the frontal plane throughout the movement cycle. This is associated with larger angle values (predominance) on the involved side than on the uninvolved side (pelvis shifted upward). The average (median) asymmetry value was around −27%, with the highest SF value (−37.4%) during the initial stance and terminal swing phase and the lowest SF value (−15.6%) at the beginning of the swing phase.

**Figure 4:**
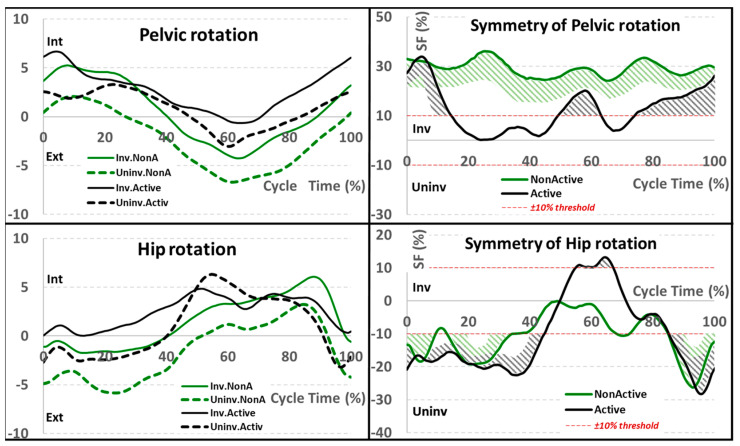
Transverse plane kinematics for pelvis and hip joints (in degrees) for both involved/uninvolved limb and active/non-active groups (**on the left**); corresponding symmetry function (SF in percent) for the active/non-active groups (**on the right**). The area exceeding the ±10% threshold was highlighted to discriminate between symmetrical and asymmetrical patterns. All angles are in degrees and the SF in percentage.

Similarly, the pelvis and the hip moved relatively asymmetrically in the transverse plane (Figure 4, Table 2), with SF values being statistically higher for the NAC patients. For pelvic internal/external rotation, the average SF values for the AC and NAC groups were 12 and 29%, respectively. The highest symmetry values were up to 34% for the AC group and 35% for the NAC group. Moreover, the NAC’s pattern was asymmetrical throughout the entire gait cycle (GC), whereas the AC’s pattern was only between 45–75% GC. The highest asymmetry for hip rotation in both groups was found during the stance phase and at terminal swing. The symmetry values were similar, ranging from −27 to 13%, with an average of −12%.

**Figure 5:**
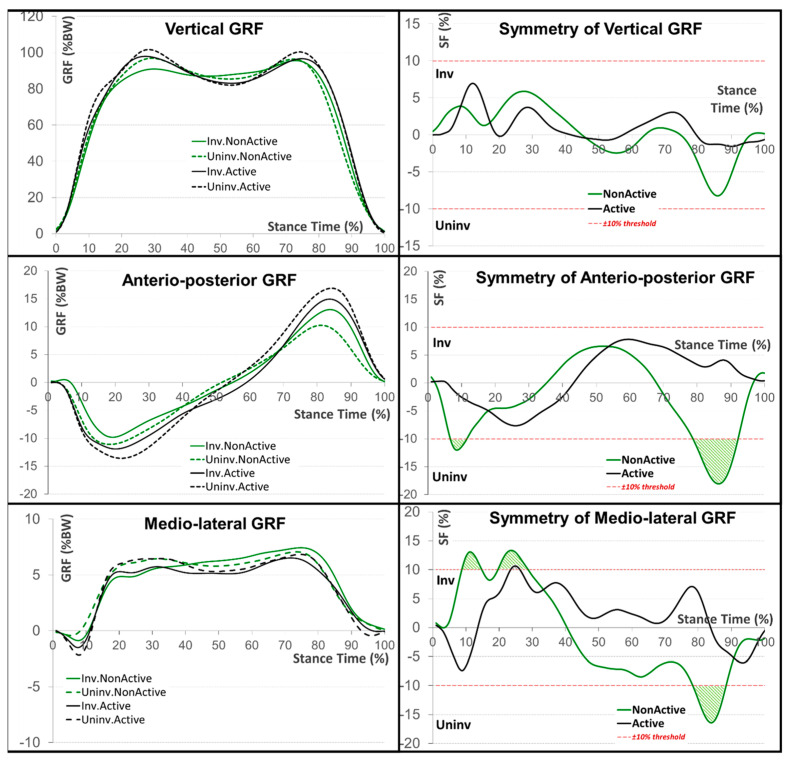
Ground reaction force components (in % of body weight) for both involved/uninvolved limbs and active/non-active groups (**on the left**); corresponding symmetry function (SF) for the active/non-active groups (**on the right**). The ±10% threshold was postulated to discriminate between symmetrical and asymmetrical patterns. All angles are in degrees and the SF in percentage.

The GRF pattern (Figure 5, Table 3) resembled the typical saddle-shaped pattern of a healthy individual. However, the values for the prosthetic-involved limb were lower, resulting in relative differences between the sides. However, these differences were not generally greater than the adopted +/−10% level; only the minimum value of SF for the AP group (a-pGRF) and mediolateral (m-lGRF) components for the NAC group resulted in −18% for a-pGRF and −16% for m-lGRF and occurred at 80–90% ST. Statistical tests also proved this phenomenon. There were significant side differences for the minimum value of a-pGRF (AC and NAC groups), the maximum value of a-pGRF (NAC group), and the minimum value of m-lGRF for both groups.

## 4. Discussion

According to the present study on a group of AKA amputees who used their prostheses on a daily basis, only 43% of them declared that they were physically active (65% walking, 42% strengthening exercises) [24]. The PA ranges for amputees do not meet WHO recommended guidelines. The AC group declared PA met WHO recommendations. Nevertheless, the gait of both AC and NAC participants was asymmetrical. Asymmetry affected both kinematic and GRF variables, especially in the AP direction. The gait of NAC participants compared to AC ones was found to be more asymmetrical, especially for the limb involved in pelvic tilt, obliquity and rotation, hip abduction/adduction, and rotation. Compared to AC participants, NAC participants moved with a more anteriorly tilted pelvis on the involved side of the limb. The hip joint and knee joint were more flexed during the swing phase on the same side. This mechanism (hip hiking) is frequently observed in individuals with lower limb joint pain or as a result of hip girdle muscle weakness (mainly the gluteus medius muscle). In NAC participants, there was a tilted pelvis in the frontal plane during the mid-swing on the uninvolved side of the limb. The thigh at the hip joint was much more adducted throughout the gait cycle on the uninvolved side of the limb. The pelvis on the same side exhibited greater internal rotation throughout the gait cycle. In contrast, the thigh exhibited greater external rotation during the stance phase in the NAC group compared to the AC group. Attempts to direct the center of body weight closer to the limb during stance (caused by pain in the involved limb or by hip girdle muscle weakness) may be considered the reason for this type of compensation. As mentioned in the introduction, OA is a common complication of AKA resulting from gait asymmetry. A similar mechanism of gait asymmetry occurs in individuals with hip OA [31]. In their study concerning PA with reduced function and pain caused by hip OA, Leichtenberg et al. [20] report no relationship between PA and OA. Similarly, Chen et al. [22] recommend moderate PA for individuals with mild OA to improve musculoskeletal function. Simic et al. [23] report that PA may be a risk-reducing factor for radiographic progression of OA within 1–2 years. According to this study, AC amputees had less asymmetrical gait.

Painful thigh stump or lumbar spine pain affects gait pattern, which contributes to the increased asymmetry of movement between the involved and uninvolved limbs [26,32]. Greater asymmetry between limbs, in turn, contributes to further exacerbation of already existing gait dysfunctions. One way to reduce pain or counteract its negative effects is by physical activity. In the study group, there were pronounced differences between the AC and NAC groups in terms of the asymmetry of the ranges of motion between involved and uninvolved limbs with respect to the analyzed kinematic parameters. The asymmetry between limbs in the AC group was observed mainly in the transverse plane of rotational movements of the pelvis and hip joint, where SF varied within 20% asymmetry; in the NAC group, the differences between limbs were more pronounced and affected all analyzed planes, with SF ranging within 60% asymmetry for pelvic motion in the sagittal, frontal, and transverse planes. Beebe et al. reported that one of the reasons for changes in lower extremity ranges of motion during gait might include adaptive changes related to both fears of pain and avoidance behaviors (greatest asymmetry in the transverse plane of hip joint motion) [9]. It should be noted that AC participants who lost a lower limb at a young age (osteosarcoma) had less asymmetry between limbs than the corresponding NAC group (congenital malformation). Krekoukias et al. [11] reported that asymmetry in the ranges of motion between the limbs of those with lower back pain affects the symmetry of pelvic motion during gait. According to Ogawa et al. [12], those with chronic pain have a slower gait speed.

### 4.1. Practical Implications

Determination of physiotherapy recommendations is a critical area in the work of any therapy team for AKA amputees. AKA amputees should be encouraged to engage in physical activity as an adjunct to rehabilitation. The recommendations should be tailored to each case individually. This study provides additional arguments for encouraging AKA amputees to engage in PA.

### 4.2. Study Limitations

This study has several limitations. Firstly, this study enrolled a relatively small number of patients. However, power analysis was performed to determine the sample size, and more patients were enrolled compared to the least necessary number of participants. Secondly, the study was conducted in the clinical setting of only one center. The data from the uninvolved side limbs were used as controls in each group. A control group of healthy participants would make the results more meaningful.

## 5. Conclusions

Despite movement asymmetries, especially within the pelvis and hip joints, AKA amputees should not avoid PA. SF discriminated areas of the highest and lowest symmetry values for both active and non-active patients. The AC’s pattern was more symmetrical than the NAC’s pattern, especially in the case of pelvic and hip joint motion. It should be noted that physical activity must be consulted with members of the medical team.

## Figures and Tables

**Table 1:** Detailed characteristics of patients’ gender, body height, mass, age, and type of prosthesis. Regular sporting activity for the active group was highlighted.

Subject	Body Height [m]	Body Mass [kg]	Age [Year]	Side	Cause	Stump Length [cm]	Socket Type	Prosthetic KNEE	Prosthetic FOOT	Sporting Activity
S1	1.71	73.2	46	R	Trauma	29.5	ICS Anatomica	C-Leg^®^	1C40 C-Walk^®^	Bodybuilding
S2	2.00	106.0	43	L	Trauma	35.0	ICS Anatomica	C-Leg^®^	1C40 C-Walk^®^	Tenis
S3	1.78	96.0	63	L	Trauma	29.0	Marlo Anatomical Socket (MAS^®^)	3R80^®^	1C30 Trias^®^	Non-active
S4	1.83	111.5	19	L	Congenital Malformation	34.0	ICS Anatomica	3R95^®^	1C30 Trias^®^	Non-active
S5	1.82	93.0	45	R	Trauma	21.0	ICS Anatomica	C-Leg^®^	1C40 C-Walk^®^	Non-active
S6	1.71	58.3	20	R	Cancer	22.0	ICS Anatomica	C-Leg^®^	1C40 C-Walk^®^	Swimming
S7	1.75	81.0	54	L	Trauma	24.0	ICS Anatomica	3R80^®^	1C30 Trias^®^	Volleyball
S8	1.68	60.0	36	L	Vascular	27.0	ICS Anatomica	3R80^®^	1C30 Trias^®^	Non-active
S9	1.64	46.4	21	L	Cancer	22.5	ICS Anatomica	C-Leg^®^	1C60 Triton	Swimming
S10	1.70	80.2	33	L	Congenital Malformation	24.0	ICS Anatomica	3R95^®^	1C30 Trias^®^	Non-active
S11	1.70	58.1	64	R	Cancer	25.5	Marlo Anatomical Socket (MAS^®^)	3R80^®^	1E56 Axtion^®^	Non-active
S12	1.83	97.0	58	L	Trauma	32.0	ICS Anatomica	C-Leg^®^	1C60 Triton^®^	Tenis
S13	1.63	59.7	38	R	Trauma	28.5	ICS Anatomica	C-Leg^®^	1C60 Triton^®^	Non-active
S14	1.81	93.6	41	R	Cancer	25.0	ICS Anatomica	C-Leg^®^	1C40 C-Walk^®^	Volleyball

**Table 2:** Sagittal, frontal and transversal plane kinematic results. Median, minimal, and maximal values and ±median absolute deviation (MAD).

			Active (AC) Group						Non-Active (NAC) Group					
		Parameter	Angle Value [deg]				SF Function [%]		Angle Value [deg]				SF Function [%]	
			Involved		Uninvolved				Involved		Uninvolved			
**Sagittal plane**	**Pelvic tilt**	Median ± MAD	12.9	±1.2 ^#^	13.1	±1.3	2.2	±1.9 ^#^	15.5	±1.2 *	12.6	±1.8	58.6	±7.8
		Min ± MAD	10.3	±1.1 ^#^	11.5	±1.9	−26.4	±5.2 ^#^	13.2	±1.1 *	10.3	±1.2	43.2	±10.1
		Max ± MAD	16.0	±3.3 ^#^	13.8	±2.7	23.8	±5.5 ^#^	17.6	±3.3 *	13.9	±2.6	76.9	±9.2
	**Hip flexion-extension**	Median ± MAD	16.4	±3.8 ^#^	15.8	±1.4 ^#^	−3.3	±2.7 ^#^	19.5	±4.8	20.7	±3.3	5.8	±5.3
		Min ± MAD	0.2	±1.3 *^,#^	−2.3	±1.2 ^#^	−7.1	±5.4	4.4	±1.8 *	0.9	±1.1	−9.7	±6.1
		Max ± MAD	31.0	±6.2	29.9	±4.7 ^#^	0.3	±3.2 ^#^	31.0	±2.4 *	34.9	±2.7	12.5	±5.8
	**Knee flexion-extension**	Median ± MAD	1.3	±1.2 *^,#^	8.1	±3.1 ^#^	5.9	±2.0	3.8	±0.7 *	9.8	±1.2	8.0	±2.3
		Min ± MAD	−1.2	±0.2 ^#^	−2.7	±1.5 ^#^	−15.4	±5.3 ^#^	1.9	±0.5 *	0.5	±0.3	−6.6	±5.4
		Max ± MAD	45.4	±5.4	44.1	±6.1	22.1	±6.3	36.9	±5.9	47.6	±3.3	29.7	±6.7
	**Ankle dorsi-plantarflexion**	Median ± MAD	1.0	±0.5 *	−1.1	±0.2	0.4	±4.2 ^#^	1.1	±0.2 *	−0.8	±0.4	−9.1	±4.2
		Min ± MAD	0.5	±9.6 *^,#^	−11.9	±0.2 ^#^	−9.9	±9.9 ^#^	−7.9	±3.1 *	−12.2	±2.2	−26.7	±8.8
		Max ± MAD	2.1	±3.2 *^,#^	4.6	±2.6 ^#^	9.8	±9.7 ^#^	6.2	±1.8 *	5.8	±1.0	−1.6	±6.1
**Frontal plane**	**Pelvic obliquity**	Median ± MAD	1.4	±1.2 ^#^	2.0	±1.1	−11.2	±9.5 ^#^	0.7	±1.2 *	2.7	±1.1	−26.7	±7.9
		Min ± MAD	−3.1	±1.1	−4.0	±0.6 ^#^	−62.3	±10.7 ^#^	−4.2	±1.1 *	−1.3	±1.4	−37.4	±9.3
		Max ± MAD	6.6	±1.3 ^#^	5.1	±1.8	67.0	±9.3 ^#^	4.5	±1.3 *	7.0	±1.9	−15.6	±6.5
	**Hip ab/adduction**	Median ± MAD	−0.2	±0.5 *^,#^	1.4	±1.0 ^#^	4.2	±5.5 ^#^	−6.9	±0.8 *	−2.2	±1.2	54.1	±7.0
		Min ± MAD	−2.3	±1.6 ^#^	−1.4	±1.1 ^#^	−12.8	±9.4 ^#^	−8.1	±1.1 *	−4.0	±1.3	39.5	±10.2
		Max ± MAD	3.6	±3.0 ^#^	2.9	±2.1 ^#^	36.2	±7.1 ^#^	−4.4	±1.4 *	0.8	±1.2	79.9	±8.9
**Transversal plane**	**Pelvic rotation**	Median ± MAD	2.9	±1.1 *^,#^	1.3	±1.7 ^#^	11.9	±3.5 ^#^	0.3	±1.4 *	−2.2	±1.4	29.1	±6.7
		Min ± MAD	−0.6	±0.3 *^,#^	−3.0	±0.5 ^#^	0.2	±4.3 ^#^	−4.3	±1.2 *	−6.7	±1.2	24.0	±8.9
		Max ± MAD	6.7	±0.5 *^,#^	3.3	±1.9 ^#^	34.0	±9.0	5.2	±1.5 *	2.1	±1.3	36.0	±9.5
	**Hip rotation**	Median ± MAD	2.8	±0.3 *^,#^	0.0	±1.0 ^#^	−16.2	±4.5	1.3	±0.6 *	−2.0	±0.7	−10.1	±7.9
		Min ± MAD	0.0	±0.2 *	−3.2	±0.6 ^#^	−28.2	±8.1 ^#^	−1.7	±0.6 *	−5.9	±0.4	−26.3	±9.1
		Max ± MAD	4.9	±1.2	6.3	±2.8 ^#^	13.2	±6.3	6.1	±1.4 *	3.2	±0.5	−0.1	±8.0

* statistically significant difference between involved and uninvolved sides, α = 0.05 ^#^ statistically significant difference between active and non-active groups, α = 0.05.

**Table 3:** GRF results. Median, minimal, and maximal values and ±median absolute deviation (±MAD).

		Active Group						Non-Active Group					
		GRF Value [N]				SF Function [%]		GRF Value [N]				SF Function [%]	
Component	Parameter	Involved		Uninvolved				Involved		Uninvolved			
Vertical	Median ± MAD	85.1	±7.4	85.3	±7.6	0.3	±0.5	87.3	±7.5	86.1	±7.6	0.5	±0.9
	Min ± MAD	1.0	±0.8	0.4	±0.8 ^#^	−1.6	±0.1 ^#^	1.4	±0.8	1.5	±0.8	−8.2	±0.1
	Max ± MAD	97.9	±7.3	101.6	±6.7	7.0	±0.5 ^#^	95.6	±7.4	96.9	±6.8	5.9	±0.8
Antero- posterior	Median ± MAD	−1.2	±2.1 ^#^	0.0	±2.4	1.1	±1.2 ^#^	0.1	±1.7	0.2	±1.7	−2.9	±1.7
	Min ± MAD	−11.9	±0.2 *^,#^	−13.6	±0.3 ^#^	−7.6	±0.1 ^#^	−9.8	±0.2 *	−11.1	±0.2	−18.0	±0.2
	Max ± MAD	14.9	±2.1	16.9	±2.4 ^#^	7.8	±1.2	13.1	±1.7 *	10.2	±1.6	6.6	±1.7
Medio- lateral	Median ± MAD	5.1	±0.6	5.5	±0.7	2.3	±1.1	5.7	±0.7	5.9	±0.6	−2.6	±2.1
	Min ± MAD	−1.5	±0.2 *^,#^	−2.2	±0.2 ^#^	−7.4	±0.3 ^#^	−0.9	±0.2 *	−0.4	±0.1	−16.4	±0.7
	Max ± MAD	6.5	±0.6	6.8	±0.7	10.6	±1.1 ^#^	7.4	±0.7	7.1	±0.6	13.3	±2.1

* statistically significant difference between involved and uninvolved sides, α = 0.05 ^#^ statistically significant difference between active and non-active groups, α = 0.05.

## Data Availability

The data relating to this study are available from the corresponding author upon reasonable request.
